# Hygrothermal Analysis of Laminated Composite Skew Conoids

**DOI:** 10.3390/ma12020225

**Published:** 2019-01-10

**Authors:** Abhay Chaubey, Ajay Kumar, Stanisław Fic, Danuta Barnat-Hunek, Barbara Sadowska-Buraczewska

**Affiliations:** 1 Department of Civil Engineering, Koneru Lakshmaiah Education Foundation, Vaddeswaram 522502, India; 2 Department of Civil Engineering, National Institute of Technology Patna, Patna 800005, India; 3 Faculty of Civil Engineering and Architecture, Lublin University of Technology, Str. Nadbystrzycka 40, 20-618 Lublin, Poland; s.fic@pollub.pl (S.F.); d.barnat-hunek@pollub.pl (D.B.-H.); 4 Faculty of Civil and Environmental Engineering, Bialystok University of Technology, Str. Wiejska 45A, 15-351 Bialystok, Poland; barbara.sadowska@pb.edu.pl

**Keywords:** hygrothermal analysis, finite element method, shell, laminated

## Abstract

The present paper is the first study on the hygrothermal analysis (i.e., effect of temperature and moisture loadings) of laminated composite skew conoids with reasonable depth and thickness. In order to solve the hygrothermal problem of laminated composite skew conoids, the cubic variation in displacement field, along with cross curvature effects of the shell, were considered. In the present analysis, the shear correction factor is not needed due to the parabolic variation of transverse shear strain. The zero transverse shear stress conditions at the top and bottom of the shell were imposed in the mathematical model. The novelty of our model is reflected by the simultaneous addition of twist curvature in the strain field, as well as the curvature in the displacement field allowing the reasonably thick and deep laminated composite rhombic conoid. The conoid behavior differs from the usual shells, like cylindrical or spherical ones, due to its inherent twist curvature with the complex geometry and different location of maximum deflection. The finite element (FE) implementation of the present realistic mathematical model was carried out using a nine-noded curved isoparametric element with seven unknowns at each node. The C^0^ FE implementation of the present mathematical model was done and coded in FORTRAN. The present model results were compared and found in good agreement with other solutions published in the literature. Hygrothermal analysis was performed for skew conoids having a different skew angle, temperature, moisture concentration, curvatures, ply orientation, thickness ratio, and boundary conditions.

## 1. Introduction

Nowadays, laminated composite structures are gaining increasing attention due to their enhanced properties, such as high strength to stiffness ratio, high strength to weight ratio, improved toughness, and resistance to oxidation and corrosion. Laminated composite conoids are structurally stiff and lightweight; thus, they can be applied to cover column-free large space in aircraft hangars, industrial structures, and large assembly halls. In their service lifespan, laminated structures are often exposed to adverse temperature and moisture loading. The changes in temperature and moisture cause deformation and stresses in laminated composite structures and hence lead to the failure of the structure. Therefore, it is important to conduct the analysis of laminated composite elliptic paraboloids under hygrothermal loading. 

For the analysis of plate or shell, classical plate theory (CPT) proposed by Kirchhoff [1] is the first theory which was implemented by numerous researchers for the analysis of thin plate or shell structures. However, CPT neglects the effects of shear deformation and further assumes that normal to the mid-plane remains straight and normal to the mid-surface after deformation. As a consequence, the CPT usually underestimates the deflection parameter and overestimates the natural frequencies and buckling loads, especially for thick plates. Additionally, this theory may be unsuitable for the structures made of thick and deeply laminated shells. The major limitation of this theory was identified during the analysis of thick plates, where the contribution of shear deformation cannot be neglected. In order to propose an alternative solution, a theory which considers the variation of shear deformation in a linear sense was introduced by Reissner-Mindlin (Mindlin, [2]; Reissner, [3]) as first-order shear deformation theory (FSDT). However, the linear assumption of shear deformation variation leads to the use of a shear correction factor in order to account for the realistic parabolic variation of transverse shear strain. As cited in the literature, these factors are very sensitive to the geometric properties, loading, and boundary conditions. As far as addressing the issues related with CPT and FSDT are concerned, many higher order shear deformation theories were proposed (Reddy [4]; Lo et al. [5]) to accomplish the realistic parabolic variation of transverse stresses through the thickness. Generally, HSDT (Higher order Shear Deformation Theory) involves the higher order term in Taylor’s expansion of the displacement component along the thickness direction.

Researchers have used various approaches to conduct the hygrothermal analysis of shell or plate. The problem of thermal flexure of an anisotropic thin plate was studied by Pell [6]. Whitney and Ashton [7] presented the effect of environment on laminated composite plates. They developed the equations for the laminated plate, which include the effect of expansional strains induced by temperature and moisture. The hygrothermal response of the laminated composite plate was studied by Pipes et al. [8]. They used the classical equation of diffusion to accurately describe the diffusion of moisture through the thickness. However, their results were limited to thin geometry. Reddy and Hsu [9] developed an FE formulation for an anisotropic composite plate subjected to mechanical and thermal loadings. They validated their results using an exact closed-form solution of a laminated composite plate subjected to sinusoidal loadings. In the same year, Wu and Tauchert [10] presented the thermal deformation and stress results in antisymmetric angle-ply and cross-ply laminates. They also validated their results by developing the exact solutions for the response of simply supported plates to general three-dimensional temperature variations. Deflections were computed to illustrate the thermoelastic behavior of laminates subjected to constant and linearly varying temperature. In order to study the hygrothermal behavior of shells, Lawrence and Doxsee [11] used a higher order theory. Their formulations are valid for the various shapes of shells as well as arbitrary moisture and temperature distributions. Lee and Yen [12] applied the FE method to study the problem of temperature and moisture effects on the cylindrical composite shell. Lee et al. [13] used CLPT (Classical Laminated Plate Theory) and von Karman larger deflection theory to study the hygrothermal effect on the cylindrical bending of symmetric angle-ply laminates under uniform transverse load. Ram and Sinha [13] used the FE method with the quadratic isoparametric element to study the hygrothermal effect on the bending behavior of laminates. A survey on the response to the thermal loading was conducted by Tauchert [14]. He discussed the thermally induced bending, buckling, post-buckling large deformation, and vibrational analysis. The thermal analysis of cross-ply shallow shells was presented by Khdeir et al. [15] using an exact analytical solution. Ali et al. [16] developed an accurate model for the thermal and mechanical analysis of thick laminates using a new displacement-based higher order theory. Zenkour and Fares [17] developed a single layer model for the thermal bending analysis of laminated cylindrical shells using FSDT. The static and dynamic behavior of thick composite laminates under hygrothermal condition was presented by Patel et al. [18] using HSDT. Khare et al. [19] presented closed form solutions for the thermo-mechanical analysis of doubly curved laminated shells using a 2D HSDT theory. A unified shear deformation plate theory was used by Zenkour [20] to study the thermo-elastic behavior of anti-symmetric and symmetric cross-ply laminates. Brischetto and Carrera [21] presented a bending analysis of a multilayered plate under thermo-mechanical loadings. Nonlinear flexural analysis of laminates subjected to hygro-thermo-mechanical loading was studied by Upadhyay et al. [22]. Lal et al. [23] presented a direct iterative based C^0^ nonlinear FE method for the plate and the spherical shell panel subjected to hygro-thermo-mechanical loading. An efficient HSDT was used by Singh and Chakrabarti [24] for the hygrothermal analysis of laminates. In the framework of the Carrera’s unified formulation, a refined 2D model was proposed by Brischetto [25] for the bending analysis of multilayered composite and sandwich shells under hygrothermal and mechanical loadings. Ali et al. [26,27] performed the hygrothermal analysis of cylindrical shells using HSDT. An experimental and a numerical study based on FSDT was presented by Biswal et al. [28] for the dynamic analysis of laminated shallow shells under hygrothermal conditions. A sinusoidal shear deformation theory was used by Zenkour and Alghanmi [29] to study the sinusoidal hygrothermal loading on multilayered plates. Jin and Yao [30] presented an efficient improved C^0^-type global-local model (IGLM) to study the bending analysis of thick cross-ply laminates under hygrothermal loadings.

Hadid [31] used a combined variational approach for simply supported and clamped elastic conoids to explore the bending response. In his mathematical formulation, he reduced displacement-based shell equations by utilizing the “Kantorovich method” into a differential equation. A modified isoparametric element was used by Choi [32] to perform the static analysis of truncated thin conoids. Ghosh and Bandyopadhyay [33] used a doubly curved quadratic isoparametric eight-node element to study the bending behavior of conoids. In 1990, Ghosh and Bandyopadhyay [34] presented an approximate static analysis of truncated conoids using “Galerkin method” in a simple form. The bending behavior of laminated parabolic conoids was presented by Dey et al. [35], using the FE method. Das and Bandyopadhyay [36] investigated the bending response of conoids using the FE analysis and Experimental analysis. The effect of cutouts on the bending behavior of conoids was presented by Ghosh and Bandyopadhyay [37], using their own formulation stated above. Das and Chakravorty [38] employed the first order shear deformation theory (FSDT) for the static analysis of laminated conoids. Their FE code was developed using the eight-node curved isoparametric elements. The bending behavior of stiffened conoids was studied by Das and Chakravorty [39], who used a beam element having three nodes in assemblage with an eight-noded shell element. Many other researchers [40,41,42,43,44] have worked on development of theory of laminated structures.

The literature survey reveals that there are no results on the hygrothermal analysis of the laminated composite skew conoids. Therefore in this paper, an attempt was made to study this phenomenon. A C^0^ FE model using a nine-noded continuous curved isoparametric element was developed by the authors for the present study.

## 2. Mathematical Formulations

### 2.1. Displacement Fields and Strains

**Figure 1:**
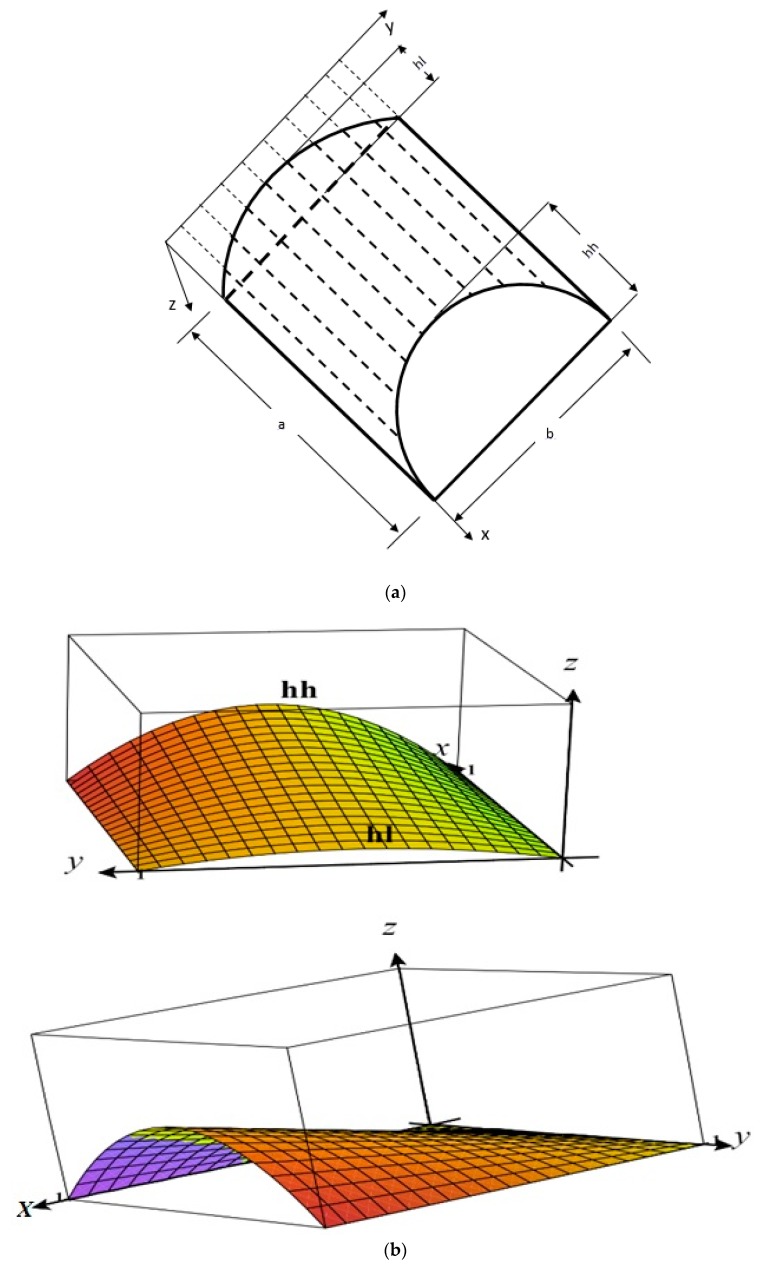

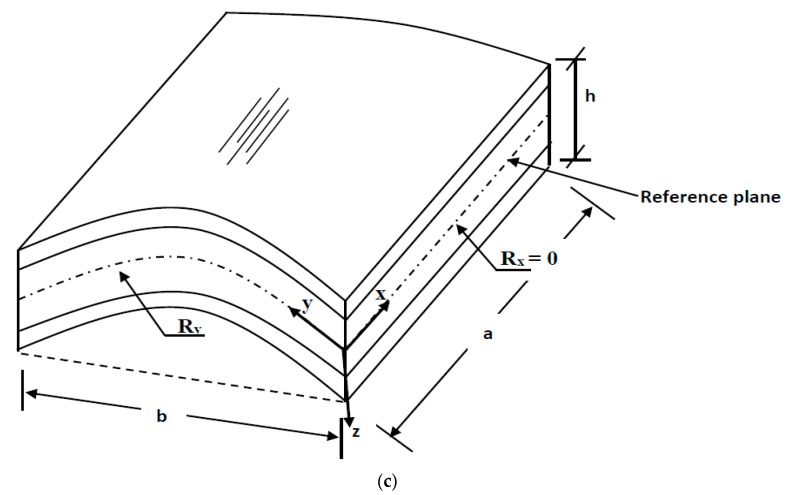
(**a**) Geometry of a conoidal shell. (**b**) 3D Geometry of a conoidal shell. (**c**) Laminated composite conoidal shell.

A composite laminated parabolic conoid having a and b sides in *x* and *y*-direction, respectively, and a uniform thickness *h* in *z*-direction is shown in Figure 1a–c. The *x* and *y* axes represent the lines of curvature and the reference plane was selected at *z* = 0. The surface equation of conoidal shell $z(x,y)=4\left[hl+(hh-hl)\frac{x}{a}\right]\left[\frac{y}{b}-\frac{y^{2}}{b^{2}}\right]$, where *hl* is the minimum rise and *hh* is the maximum rise of the conoidal shell.

Curvature $\frac{1}{R_{x}}=\frac{\partial^{2}z}{\partial x^{2}}$, $\frac{1}{R_{y}}=\frac{\partial^{2}z}{\partial y^{2}}$ and cross curvature $\frac{1}{R_{xy}}=\frac{\partial^{2}z(x,y)}{\partial x\partial y}$ (i.e., twist of the surface with respect to *x* and *y* directions) were used in the model. The present theory is suitable for shallow and moderately thick conoidal shells. 

The enhanced displacement field with curvature effect can be expressed as:

(1) $$\begin{array}{l} u(x,y,z)=\left(1+\frac{z}{R_{x}}\right)u_{0}(x,y)+z\theta_{x}(x,y)+z^{2}\xi_{x}(x,y)+z^{3}\zeta_{x}(x,y),\\ v(x,y,z)=\left(1+\frac{z}{R_{y}}\right)v_{0}(x,y)+z\theta_{y}(x,y)+z^{2}\xi_{y}(x,y)+z^{3}\zeta_{y}(x,y),\\ w(x,y,z)=w_{0}(x,y) \end{array}\}$$

where *R_x_* and *R_y_* are curvatures along *x* and *y*-direction.

Displacement components $u_{0}$, $v_{0}$, and $w_{0}$ are the translations at the reference plane of the conoids. Rotation to the normal reference plane about *y* and *x*-axis are $\theta_{x}(x,y)$ and $\theta_{y}(x,y)$. The $\xi_{x}$, $\zeta_{x}$, $\xi_{y}$, and $\zeta_{y}$ are higher-order terms that can be found using the zero transverse stress conditions at the top and bottom of conoids.

The enhanced strain-displacement relationships with the cross curvature effect are expressed as:

(2) $$\begin{array}{l} \varepsilon_{x}=\frac{\partial u}{\partial x}+\frac{w}{R_{x}}\\ \varepsilon_{y}=\frac{\partial v}{\partial y}+\frac{w}{R_{y}}\\ \gamma_{xy}=\frac{\partial v}{\partial x}+\frac{\partial u}{\partial y}+\frac{2w}{R_{xy}}\\ \gamma_{xz}=\frac{\partial u}{\partial z}+\frac{\partial w}{\partial x}-\frac{u_{0}}{R_{x}}-\underline{\frac{v_{0}}{R_{xy}}}\\ \gamma_{yz}=\frac{\partial v}{\partial z}+\frac{\partial w}{\partial y}-\frac{v_{0}}{R_{y}}-\underline{\frac{u_{0}}{R_{xy}}} \end{array}\}$$

where $\frac{1}{R_{xy}}$ is the cross curvature of the shell.

The zero shear force condition at the free surface:

(3) $$\sigma_{xz}\left(x,y,\pm \frac{h}{2}\right)=0\;i.e.,\;Q_{44}\gamma_{xz}\left(x,y,\pm \frac{h}{2}\right)+Q_{45}\gamma_{yz}\left(x,y,\pm \frac{h}{2}\right)=0,$$

and

(4) $$\sigma_{yz}\left(x,y,\pm \frac{h}{2}\right)=0\;i.e.,\;Q_{45}\gamma_{xz}\left(x,y,\pm \frac{h}{2}\right)+Q_{55}\gamma_{yz}\left(x,y,\pm \frac{h}{2}\right)=0,$$

where $Q_{ij}$ are the material constants.

From Equations (3) and (4), we get:

$$\gamma_{xz}\left(x,y,\pm \frac{h}{2}\right)=\gamma_{yz}\left(x,y,\pm \frac{h}{2}\right)=0$$

Now the condition $\gamma_{xz}\left(x,y,\pm \frac{h}{2}\right)=0$ yields the following:

(5) $$\theta_{x}+\frac{\partial w_{0}}{\partial x}-\frac{v_{0}}{R_{xy}}+\frac{h}{2}(2\xi_{x})+\frac{3h^{2}}{4}(\zeta_{x})=0$$

(6) $$\theta_{x}+\frac{\partial w_{0}}{\partial x}-\frac{v_{0}}{R_{xy}}-\frac{h}{2}(2\xi_{x})+\frac{3h^{2}}{4}(\zeta_{x})=0$$

From Equations (5) and (6):

(7) $$\xi_{x}=0\;\mathrm{and}\;\zeta_{x}=-\frac{4}{3h^{2}}\left[\frac{\partial w_{0}}{\partial x}+\theta_{x}-\frac{v_{0}}{R_{xy}}\right]$$

Similarly, from $\gamma_{yz}\left(x,y,\pm \frac{h}{2}\right)=0$ we get:

(8) $$\xi_{y}=0\;\mathrm{and}\;\zeta_{y}=-\frac{4}{3h^{2}}\left[\frac{\partial w_{0}}{\partial y}+\theta_{y}-\frac{u_{0}}{R_{xy}}\right]$$

Substituting Equations (7) and (8) in Equation (1), we get:

(9) $$\begin{array}{l} u(x,y,z)=\left(1+\frac{z}{R_{x}}\right)u_{0}+\theta_{x}\left(z-\frac{4z^{3}}{3h^{2}}\right)+\psi_{x}\left(-\frac{4z^{3}}{3h^{2}}\right)+v_{0}\left(\frac{4z^{3}}{3h^{2}R_{xy}}\right)\\ v(x,y,z)=\left(1+\frac{z}{R_{y}}\right)v_{0}+\theta_{y}\left(z-\frac{4z^{3}}{3h^{2}}\right)+\psi_{y}\left(-\frac{4z^{3}}{3h^{2}}\right)+u_{0}\left(\frac{4z^{3}}{3h^{2}R_{xy}}\right)\\ w(x,y,z)=w_{0}(x,y) \end{array}\}$$

In-plane displacements contain the $\frac{\partial w_{0}}{\partial x}$ and $\frac{\partial w_{0}}{\partial y}$ terms that are expressed in terms of the independent $\psi_{x}$ and $\psi_{y}$ variables, respectively, to avoid the difficulty associated with C^1^ continuity and to make it C^0^ continuity. 

The strain component can be generalized as:

(10) $$\begin{array}{l} \left\{\bar{\varepsilon }\right\}=\left\{\begin{array}{c} \frac{\partial u}{\partial x}+\frac{w}{R_{x}}\\ \frac{\partial v}{\partial y}+\frac{w}{R_{y}}\\ \frac{\partial v}{\partial x}+\frac{\partial u}{\partial y}+\frac{2w}{R_{xy}}\\ \frac{\partial u}{\partial z}+\frac{\partial w}{\partial x}-\frac{u_{0}}{R_{x}}-\underline{\frac{v_{0}}{R_{xy}}}\\ \frac{\partial v}{\partial z}+\frac{\partial w}{\partial y}-\frac{v_{0}}{R_{y}}-\underline{\frac{u_{0}}{R_{xy}}} \end{array}\right\}=\\ \left\{\begin{array}{c} \left(1+\frac{z}{R_{x}}\right)\varepsilon_{x0}+\kappa_{x}\left(z-\frac{4z^{3}}{3h^{2}}\right)+\kappa_{x}^{*}\left(-\frac{4z^{3}}{3h^{2}}\right)+\gamma_{x0}\left(\frac{4z^{3}}{3h^{2}R_{xy}}\right)+\frac{w_{0}}{R_{x}}\\ \left(1+\frac{z}{R_{y}}\right)\varepsilon_{y0}+\kappa_{y}\left(z-\frac{4z^{3}}{3h^{2}}\right)+\kappa_{y}^{*}\left(-\frac{4z^{3}}{3h^{2}}\right)+\gamma_{y0}\left(\frac{4z^{3}}{3h^{2}R_{xy}}\right)+\frac{w_{0}}{R_{y}}\\ \left(1+\frac{z}{R_{y}}\right)\gamma_{x0}+K_{xy}\left(z-\frac{4z^{3}}{3h^{2}}\right)+K_{xy}^{*}\left(-\frac{4z^{3}}{3h^{2}}\right)+\left(\varepsilon_{xo}+\varepsilon_{yo}\right)\left(\frac{4z^{3}}{3h^{2}R_{xy}}\right)+\left(1+\frac{z}{R_{x}}\right)\gamma_{y0}+\frac{2w_{0}}{R_{xy}}\\ \theta_{x}\left(1-\frac{4z^{2}}{h^{2}}\right)+\psi_{x}\left(1-\frac{4z^{2}}{h^{2}}\right)+v_{0}\left(\frac{4z^{2}}{h^{2}R_{xy}}-\frac{1}{R_{xy}}\right)\\ \theta_{y}\left(1-\frac{4z^{2}}{h^{2}}\right)+\psi_{y}\left(1-\frac{4z^{2}}{h^{2}}\right)+u_{0}\left(\frac{4z^{2}}{h^{2}R_{xy}}-\frac{1}{R_{xy}}\right) \end{array}\right\} \end{array}$$

where

$$\begin{array}{l} \varepsilon_{x0}=\frac{\partial u_{0}}{\partial x},\varepsilon_{y0}=\frac{\partial v_{0}}{\partial y},\gamma_{x0}=\frac{\partial v_{0}}{\partial x}\\ \kappa_{x}=\frac{\partial \theta_{x}}{\partial x},\kappa_{y}=\frac{\partial \theta_{y}}{\partial y},\gamma_{y0}=-\frac{\partial u_{0}}{\partial y}\\ \kappa_{x}^{*}=\frac{\partial \psi_{x}}{\partial x},\kappa_{y}^{*}=\frac{\partial \psi_{y}}{\partial y},\kappa_{xy}=\left(\frac{\partial \theta_{y}}{\partial x}+\frac{\partial \theta_{x}}{\partial y}\right),\kappa_{xy}^{*}=\left(\frac{\partial \psi_{y}}{\partial x}+\frac{\partial \psi_{x}}{\partial y}\right) \end{array}$$

The strains associated with Equation (10) are related to the generalized strains by the means of the following expression:

(11) $$\left\{\bar{\varepsilon }\right\}=\left[H\right]\left\{\varepsilon \right\}$$

where $\left\{\bar{\varepsilon }\right\}={\left[\varepsilon_{x}\varepsilon_{y}\gamma_{xy}\gamma_{xz}\gamma_{yz}\right]}^{T}$ and $\left\{\varepsilon \right\}={\left\{\begin{array}{l} \varepsilon_{x0},\kappa_{x},\kappa_{x}^{*},\gamma_{x0},\varepsilon_{y0},\kappa_{y},\kappa_{y}^{*},\gamma_{y0},\\ \kappa_{xy},\kappa_{xy}^{*},w_{0},\theta_{x},\psi_{x},v_{0},\theta_{y},\psi_{y},u_{0} \end{array}\right\}}^{T}$; [H] is the matrix of order 5 × 17 containing the terms involving z and h.

Further, the strain vector {ε} can be interrelated with the displacement vector {*d*} by means of the following relationship:

(12) {ε}=[B]{d}

where $\left\{d\right\}=\left\{u_{0},v_{0},w_{0},\theta_{x},\theta_{y},\psi_{x},\psi_{y}\right\}$, [*B*] is the differential operator matrix of the interpolation function which can be derived from Equation (10).

### 2.2. Constitutive Equation

For a shell of constant thickness h and composed of thin layers of orthotropic material, the constitutive equations can be derived as:

(13) $${\left\{\begin{array}{c} \sigma_{x}\\ \sigma_{y}\\ \tau_{xy}\\ \tau_{yz}\\ \tau_{xz} \end{array}\right\}}_{k}={\left[\begin{array}{ccccc} {\bar{Q}}_{11} & {\bar{Q}}_{12} & {\bar{Q}}_{16} & 0 & 0\\ {\bar{Q}}_{12} & {\bar{Q}}_{22} & {\bar{Q}}_{26} & 0 & 0\\ {\bar{Q}}_{16} & {\bar{Q}}_{26} & {\bar{Q}}_{66} & 0 & 0\\ 0 & 0 & 0 & {\bar{Q}}_{44} & {\bar{Q}}_{45}\\ 0 & 0 & 0 & {\bar{Q}}_{45} & {\bar{Q}}_{55} \end{array}\right]}_{k}{\left\{\begin{array}{c} \varepsilon_{x}-\alpha_{x}\Delta T-\beta_{x}\Delta C\\ \varepsilon_{y}-\alpha_{y}\Delta T-\beta_{y}\Delta C\\ \gamma_{xy}-\alpha_{xy}\Delta T-\beta_{xy}\Delta C\\ \gamma_{yz}\\ \gamma_{xz} \end{array}\right\}}_{k}$$

where

Transformed reduced stiffness matrix $\left[{\bar{Q}}_{ij}\right]$ can be formed with the material properties.
($E_{1},E_{2},\mu_{12},G_{12},G_{13},G_{23}$) and fiber orientation (θ) of lamina [45].
Δ*T* = Change in temperature.
Δ*C* = Change in moisture concentration.
$\beta_{x}$; $\beta_{y}$; $\beta_{xy}$ = transformed swelling or contraction coefficients due to moisture.
$\alpha_{x}$; $\alpha_{y}$; $\alpha_{xy}$ = transformed thermal expansion or contraction coefficients due to temperature.

## 3. Finite Element Formulations

A nine-noded isoparametric element was used for the present analysis.

(14) {w}=[N]{δ}

Stress at any point can be found using consecutive equation for hygrothermal analysis (Equation (6))

(15) $$\left\{\bar{\sigma }\right\}=\left[\bar{Q}\right]\left\{\bar{\varepsilon_{n}}\right\}$$

where

$$\left\{\bar{\varepsilon_{n}}\right\}=\left\{\bar{\varepsilon }-\bar{\varepsilon_{th}}-\bar{\varepsilon_{m}}\right\}$$

Now $\varepsilon_{n}$ is total strain

(16) $$\left\{\bar{\varepsilon }\right\}=\left[H\right]\left\{\varepsilon \right\}$$

where ε is strain due to mechanical loadings; $\varepsilon_{th}$ is strain due to thermal loadings; $\varepsilon_{m}$ is strain due to moisture.

(17) {ε}=[B]{d}

where {d} is the vector of nodal displacement.

In an FE formulation, the displacement and temperatures and moistures are interpolated within the domain of the element using the same interpolations functions.

By applying the virtual work method and equating the work done by internal forces we get

(18) [K]{δ}={P}

where [K] is the element stiffness matrix and {P} is nodal load vector.

(19) $$\left\{P\right\}={\iint \left[N\right]}^{T}qdxdy$$

Thermal loading is obtained by the following Equation.

(20) $$\left\{Pe^{N}\right\}={\iint \left[B\right]}^{T}{\left[H\right]}^{T}\left\{F^{N}\right\}dxdy$$

(21) $${\left\{F^{N}\right\}}^{T}=\left[N_{x}^{N},N_{y}^{N},N_{xy}^{N},M_{x}^{N},M_{y}^{N},M_{xy}^{N},0,0,---\right]$$

(22) $${\left\{N_{x}^{N},N_{y}^{N},N_{xy}^{N}\right\}}^{T}={\sum_{k}^{n}\int_{z_{k-1}}^{z_{k}}\left\{{\bar{Q}}_{ij}\right\}}_{k}{\left\{\varepsilon \right\}}_{k}dz$$

(23) $${\left\{M_{x}^{N},M_{y}^{N},M_{xy}^{N}\right\}}^{T}={\sum_{k}^{n}\int_{z_{k-1}}^{z_{k}}\left\{{\bar{Q}}_{ij}\right\}}_{k}{\left\{\varepsilon \right\}}_{k}$$

Here *i*, *j* = 1, 2, 6. Also, ${\left\{\varepsilon_{k}\right\}}^{T}=\left[\varepsilon_{x},\varepsilon_{y},\varepsilon_{xy}\right]$.

An arbitrary temperature distribution can be assumed without loss of generality of the form

(24) $$T\left(x,y,z\right)=T_{0}+\left(\frac{z}{h}\right)T_{1}(x,y)$$

where *T*_0_ is the initial constant temperature.

Case 1. When temperature is uniform across the depth

(25) $$\begin{array}{l} \left\{\begin{array}{c} \varepsilon_{x}\\ \varepsilon_{y}\\ \varepsilon_{xy} \end{array}\right\}=\left\{\begin{array}{c} \alpha_{x}\\ \alpha_{y}\\ \alpha_{xy} \end{array}\right\}\Delta T\\ \left\{\begin{array}{c} \alpha_{x}\\ \alpha_{y}\\ \alpha_{xy} \end{array}\right\}=\left[\begin{array}{ccc} c^{2} & s^{2} & -2cs\\ s^{2} & c^{2} & 2cs\\ cs & -cs & c^{2}-s^{2} \end{array}\right]{\left[Q\right]}_{k}\left\{\begin{array}{c} \alpha_{1}\\ \alpha_{2}\\ \alpha_{12} \end{array}\right\} \end{array}$$

where $\alpha_{1}$, $\alpha_{2}$, and $\alpha_{12}$ are the coefficients of thermal expansion referred to the principal material axes of the lamina and $\alpha_{x}$, $\alpha_{y}$, and $\alpha_{xy}$ are the transformed coefficients of thermal expansion referred to the *x*-*y* coordinate system.

(26) $$\left\{F\right\}=∭{\left[B\right]}^{T}{\left[H\right]}^{T}\left\{\begin{array}{c} \alpha_{x}\\ \alpha_{y}\\ \alpha_{xy} \end{array}\right\}Tdv$$

Case 2. When temperature is varying across the depth

(27) {F}=∭[B]T[H]T{αxαyαxy}[12(TU+TL+zh(TU−T))]Tdv

where $T_{U}$ = Temperature at the top surface and $T_{L}$ = Temperature at the bottom surface.

### Skew Transformations

**Figure 2:**
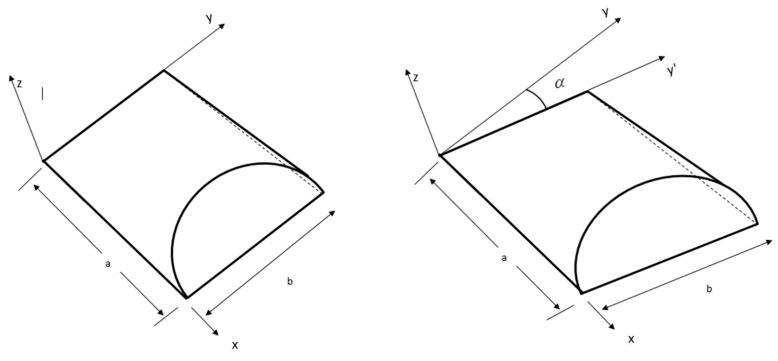
Geometry of regular conoids and distorted geometry of a skew conoid.

It is not possible to specify the boundary conditions in terms of the global displacements $u_{0},v_{0},w_{0}$, etc., because the edges of the boundary elements of skew shells (Figure 2) are not parallel to the global axis (*x*, *y,* and *z*).

Hence, to specify the required boundary conditions at the skew edge, it is necessary to use the edge displacements $u_{0}^{l},v_{0}^{l},w_{0}^{l}$ in the local coordinates $x^{l},y^{l},z^{l}$. Thus, it was mandatory to transform the element matrix corresponding to the global axis to the local axis. The transformation between the local and global axes was done using a simple transformation rule and could be expressed as:

(28) $$d_{i}=[T]d_{i}^{l}$$

where $d_{i}^{l}$ and $d_{i}$ are the generalized displacement vectors in the local and global coordinate systems of node I, respectively defined as:

(29) $$\left\{d_{i}\right\}={\left\{u_{0},v_{0},w_{0},\theta_{x},\theta_{y},\psi_{x},\psi_{y}\right\}}^{T}$$

(30) $$\left\{d_{i}^{l}\right\}={\left\{u_{0}^{l},v_{0}^{l},w_{0}^{l},\theta_{x}^{l},\theta_{y}^{l},\psi_{x}^{l},\psi_{y}^{l}\right\}}^{T}$$

Therefore, the nodal transformation matrix on the skew boundary may be presented as:

(31) $$\mathrm{Transformation}\;\mathrm{matrix}\;\left[T\right]\;=\left(\begin{array}{ccccccc} \cos\alpha & -\sin\alpha & 0 & 0 & 0 & 0 & 0\\ \sin\alpha & \cos\alpha & 0 & 0 & 0 & 0 & 0\\ 0 & 0 & 1 & 0 & 0 & 0 & 0\\ 0 & 0 & 0 & \cos\alpha & -\sin\alpha & 0 & 0\\ 0 & 0 & 0 & \sin\alpha & \cos\alpha & 0 & 0\\ 0 & 0 & 0 & 0 & 0 & \cos\alpha & -\sin\alpha \\ 0 & 0 & 0 & 0 & 0 & \sin\alpha & \cos\alpha \end{array}\right)$$

## 4. Results and Discussion

The following boundary conditions were used in the presented analysis:

- Simply supported (SSSS):
  At x=0,a; $v=w=\theta_{y}=\psi_{y}=0$
  At y=0,b; $u=w=\theta_{x}=\psi_{x}=0$
- Clamped (CCCC):
  At x=0,a and y=0,b; $u=v=w=\theta_{x}=\theta_{y}=\psi_{x}^{}=\psi_{y}^{}=0$
- Clamped-simply supported (CSCS):
  At x=0 and y=0; $u=v=w=\theta_{x}=\theta_{y}=\psi_{x}^{}=\psi_{y}^{}=0$
  At x=a; $v=w=\theta_{y}=\psi_{y}^{}=0$ and at y=b; $u=w=\theta_{x}=\psi_{x}^{}=0$

“C” represents the clamped boundary conditions, “S” represents the simply supported boundary conditions, and “F” represents the free boundary conditions.

The non-dimensional formulae used in this paper are:

$$\bar{w}=\frac{10wh}{\alpha_{0}T_{0}a^{2}},\;{\bar{\sigma }}_{x}=\left(\frac{a}{2},\frac{b}{2},-\frac{h}{2}\right)\frac{\sigma_{x}}{\alpha_{0}T_{0}E_{T}},\;{\bar{\sigma }}_{y}=\left(\frac{a}{2},\frac{b}{2},z\right)\frac{\sigma_{y}}{\alpha_{0}T_{0}E_{T}},\;\mathrm{and}\;{\bar{\tau }}_{xy}=\left(0,0,-\frac{h}{2}\right)\frac{\tau_{xy}}{\alpha_{0}T_{0}E_{T}}$$

The material properties used in this paper are: *E*_1_/*E*_2_ = 25, *G*_12_ = *G*_13_ = 0.5*E*_2_, *G*_23_ = 0.2*E*_2_, *µ*_12_ = 0.25, *ρ* = 1, and *α*_1_/*α*_2_ = 3 unless mentioned otherwise.

### 4.1. Convergence Study

A convergence study was performed by varying the mesh size (*N_x_* × *N_y_*) from 8 × 8 to 20 × 20, where *N_x_* and *N_y_* are numbers of elements in *x* and *y*-direction. Table 1 shows that the values of non-dimensional deflection of laminates (0°/90°/0°) converged for the *N_x_* × *N_y_* = 16 × 16. All the consecutive analyses were carried out with the 16 × 16 mesh size.

### 4.2. Comparison Study

Non-dimensional central deflection and stresses of laminates are compared with the 3D elastic solution of Pagano [46] and Chakrabarti and Sheikh [47]. Table 1 reveals that the present results are closer to the 3D results than the results of Chakrabarti and Sheikh [47]. Central deflection (mm) of a two-layered (0°/90°), simply-supported square laminate with different thickness ratios was presented in Table 2. The present results are in good agreement with the ones obtained by Brischetto and Carrera [21]. Table 3 shows the central deflection of SSSS rectangular laminated plates subjected to sinusoidal temperature gradient (thickness ratio (*h*/*a*) = 0.01). The present findings were validated with the results of Singh and Chakrabarti [24], Prathap and Naganarayana [48], Reddy and Hsu [9], and NASTRAN. 

The non-dimensional central deflections of three-layer (0°/90°/0°) laminates under sinusoidal temperature or hygrothermal distribution (Δ*T* = 300 °C, Δ*C* = 0.01%) are shown in Table 4. Remarkably, the obtained numerical results are in line with the ones obtained by Zenkour and Alghanmi [29]. 

In Table 5, the proposed model was validated with the deflection value of isotropic conoids along *y*/*b* = 0.5. It was observed that the results of our calculations are closer to the experimental results by Hadid [31] than the FSDT results (after Das and Chakravorty) [38]. The authors also validated their model for the composite conoids. Table 6 shows that the results obtained by the present approach are better than the ones reported by Das and Chakravorty [38] (based on FSDT).

### 4.3. Parametric Study

In this section, many new results were calculated based on the developed FE model. The dimensionless maximum deflections of three-layered laminated composite skew conoids (*hl*/*hh* = 0.25) subjected to sinusoidal hygrothermal loading were presented in Table 7. It can be seen that along with an increase in the *hl*/*hh* ratio of the conoids, the value of dimensionless maximum deflection increases as well. This is because of a reduction of shell stiffness occurring due to a decrease in shell action (as one curved edge in converting into straight). The maximum dimensionless deflection increases for the 30° and 45° skew angles and decreases for the 15° and 60° skew angles. It can be noticed that the location of maximum deflection depends upon the *hl/hh* ratio and skew angle, unlike in other shells. Table 8 shows the effect of boundary conditions on a maximum deflection of skew conoids subjected to hygrothermal loading. The maximum non-dimensional deflection was noticed for the CCFF boundary conditions and minimum deflection was found for the CCSS shell, among all the considered boundary conditions. The location of maximum deflection is also dependent upon the boundary conditions. Table 9 shows the effect of lamination angle on a non-dimensional deflection of laminated composite conoids subjected to a hygrothermal loading. It can be noticed that non-dimensional maximum deflection decreases as the number of layers increase. It can be noticed that with an increase in skew angle, the non-dimensional maximum deflection decreases for a moderately thick shell (*a*/*h* = 10). The value of maximum non-dimensional deflection of skew conoids with various side to thickness ratios was presented in Table 10. It can be observed that with an increase in the thickness of the shell, the value of maximum dimensionless deflection for all the considered skew angles increases as well. The non-dimensional maximum stresses of laminated composite conoids are shown in Table 11. It was noticed that non-dimensional stresses decrease along with the *hl*/*hh* ratio.

## 5. Conclusions

A C^0^ finite element (FE) formulation using Sanders’ approximations was developed and used to study the hygrothermal response of composite skew conoids. Numerous novel results were produced for the hygrothermal (Temperature and Moisture) response of laminated conoids having different skew angles, temperature, moisture concentrations, radii of curvatures, thickness ratios, ply orientation, and boundary conditions, which should be beneficial for a future study.

The following general conclusions can be drawn:

- The value of non-dimensional deflection of conoids under hygrothermal loadings increases along with the *hl*/*hh* ratio.
- The value of non-dimensional deflection of moderately thick shell under hygrothermal loadings decreases with an increase in the skew angle.
- The non-dimensional deflection of shells under temperature and moisture loading decreases with the increase in the number of plies.
- The non-dimensional maximum deflection of skew conoids under hygrothermal loading increases with the decreases in the *a*/*h* ratio.

## Figures and Tables

**Table 1:** Non-dimensional deflection and stresses of a SSSS square laminate (0°/90°/0°) under sinusoidal loading of amplitude *q*. (Geometric properties: *a*/*b* = 1, *a*/*h* = 100 and material properties: *E*_1_/*E*_2_ = 25, *G*_12_ = *G*_13_ = 0.5*E*_2_, *G*_23_ = 0.2*E*_2_, *µ*_12_ = 0.25).

h/a	Reference	Theory	$\bar{W}$	$\bar{\sigma_{x}}$	$\bar{\sigma_{y}}$	$\bar{\tau_{xy}}$
0.10	Present (8 × 8)		0.7174	0.5917	0.5544	0.0285
	Present (12 × 12)		0.7175	0.5880	0.5511	0.0282
	Present (16 × 16)		0.7176	0.5866	0.5499	0.0282
	Present (20 × 20)		0.7176	0.5866	0.5498	0.0280
	Pagano [46]	3D Elasticity	0.7405	0.5900		
	Chakrabarti and Sheikh [47]	HSDT	0.7140	0.5806	0.2722	0.0279
	Chakrabarti and Sheikh [47]	FSDT	0.6700	0.5219	0.2582	0.0254

**Table 2:** In-plane displacements and transverse displacement of two-layered (0°/90°), simply-supported square laminate. (Material properties: *E*_1_ = 172 GPa, *E*_2_ = 6.909 GPa, *G*_12_ = *G*_13_ = 3.45 GPa, *G*_23_ = 1.38 GPa, *µ*_12_ = 0.25)**.**

Thickness Ratio	Brischetto and Carrera [21]	Present
100	5.9448	5.9424
50	1.4857	1.4857
10	0.0587	0.0590
5	0.0141	0.0144

**Table 3:** Effect of aspect ratio on the deflection w for SSSS composite plates subjected to sinusoidal temperature gradient (thickness ratio (*h*/*a*) = 0.01) (Thickness ratio (*h*/*a*) = 0.01 and material properties: *E*_1_/*E*_2_ = 25, *G*_12_ = *G*_13_ = 0.5*E*_2_, *G*_23_ = 0.2*E*_2_, *µ*_12_ = 0.25)**.**

Reference	0°/90°/0°			0°	0°/90°
	*a*/*b* = 1	*a*/*b* = 1.5	*a*/*b* = 2	*a*/*b* = 1	*a*/*b* = 1
Present	1.0174	0.8585	0.6319	1.0226	1.1080
Singh and Chakrabarti [24]	1.0429	0.8802	0.6566	1.0332	1.1520
Prathap and Naganarayana [48]	1.0249	0.8802	0.6566	1.0332	1.1434
NASTRAN [24]	1.0028	0.8346	0.6108	1.0109	1.9374
Reddy and Hsu [9]	1.0949	0.9847	0.7643	1.0313	1.6765

**Table 4:** The validation of dimensionless center deflections of three-layer (0°/90°/0°) rectangular plates subjected to sinusoidal hygrothermal distribution (Δ*T* = 300 °C, Δ*C* = 0.01%).

*a*/*b*	*a*/*h* = 10		*a/h* = 20		*a/h* = 50	
	Present	Zenkour and Alghanmi [29]	Present	Zenkour and Alghanmi [29]	Present	Zenkour and Alghanmi [29]
1	2.7360	2.7749	2.1691	2.3654	1.9906	2.2355
1.5	3.2156	3.2273	2.9160	2.8521	2.8168	2.7148
2	3.0517	2.8496	2.9415	2.6631	2.8971	2.5849

**Table 5:** Validation of deflection (*W* × 10^−2^) of isotropic conoid subjected to uniformly distributed load along *y*/*b* = 0.50. (Geometric properties: *a* = 95 in, *b* = 95 in, *hh* = 18.0 in, *hl* = 9.0 in, *h* = 05 in, Material properties: *E* = 5,620,000 psi, *n* = 0.15, Loading: *q* = 60 psf).

*x*/*a*	Das and Chakravorty ^1^ [38]	Hadid ^2^ [31]	Present
0.10	0.9142	0.9857	0.9694
0.40	2.0428	1.8285	1.8146
0.60	1.6000	1.4000	1.4771
0.70	1.3571	1.3000	1.2637
0.80	0.9714	1.0000	0.9946

Note: ^1^ = FSDT, ^2^ = Experimental.

**Table 6:** Comparison study for maximum non-dimensional deflection ($\bar{W}$ × 10^4^) in downward direction for different laminations of conoid. (Geometric properties: *a*/*b* = 1, *a*/*h* = 100, *hl*/*hh* = 0.25. and material properties: *E*_1_/*E*_2_ = 25, *G*_12_ = *G*_13_ = 0.5*E*_2_, *G*_23_ = 0.2*E*_2_, *µ*_12_ = 0.25).

Lamination (°)	Maximum Non-Dimensional Downward Deflection $\left(\bar{W}\times 10^{4}\right)$	
	Present Theory	Das and Chakravorty [38]
0/90	0.305 (0.20, 0.50)	0.319 (0.19, 0.50)
0/90/0	0.314 (0.30, 0.50)	0.298 (0.13, 0.50)
45/−45	0.696 (0.25, 0.50)	0.722 (0.25, 0.50)
45/−45/45	0.610 (0.27, 0.40)	0.629 (0.25, 0.38)

Note: (*x*, *y*) represents the maximum deflection in downward direction.

**Table 7:** Effect of curvature (*hl*/*hh*) on the dimensionless maximum deflections of three-layer (0°/90°/0°) skew conoids subjected to sinusoidal hygrothermal distribution (Δ*T* = 300 °C, Δ*C* = 0.01%).

Skew Angle	*hl*/*hh*					
	0.25	0.20	0.15	0.10	0.05	0.00
0°	0.242 (0.40, 0.50)	0.259 (0.38, 0.50)	0.279 (0.35, 0.50)	0.306 (0.33, 0.50)	0.341 (0.28, 0.50)	0.386 (0.28, 0.50)
15°	0.203 (0.44, 0.53)	0.215 (0.44, 0.51)	0.229 (0.41, 0.51)	0.245 (0.41, 0.51)	0.265 (0.39, 0.51)	0.288 (0.39, 0.51)
30°	0.359 (0.54, 0.67)	0.352 (0.54, 0.67)	0.343 (0.54, 0.67)	0.333 (0.54, 0.67)	0.323 (0.54, 0.67)	0.312 (0.54, 0.67)
45°	0.384 (0.74, 0.57)	0.381 (0.74, 0.57)	0.378 (0.74, 0.57)	0.373 (0.73, 0.58)	0.368 (0.73, 0.58)	0.363 (0.73, 0.58)
60°	0.220 (0.91, 0.43)	0.221 (0.91, 0.43)	0.222 (0.91, 0.43)	0.223 (0.91, 0.43)	0.223 (0.91, 0.43)	0.222 (0.91, 0.43)

Note: (*x*, *y*) represents the maximum deflection in downward direction.

**Table 8:** Effect of boundary condition on the dimensionless maximum deflections of three-layer (0°/90°/0°) skew conoids subjected to sinusoidal hygrothermal distribution (Δ*T* = 300 °C, Δ*C* = 0.01%).

Skew Angle	Boundary Conditions					
	SSSS	CCCC	CSCS	CCSS	CCFF	CFCF
0°	0.242 (0.40, 0.50)	0.231 (0.40, 0.50)	0.231 (0.43, 0.50)	0.226 (0.40, 0.50)	0.615 (0.43, 0.50)	0.471 (1.00, 0.28)
15°	0.204 (0.44, 0.53)	0.209 (0.42, 0.43)	0.201 (0.42, 0.46)	0.203 (0.42, 0.43)	0.471 (0.45, 0.39)	0.882 (1.07, 0.27)
30°	0.360 (0.54, 0.67)	0.165 (0.45, 0.35)	0.165 (1.30, 0.74)	0.158 (0.45, 0.35)	0.442 (1.25, 0.87)	1.444 (1.16, 0.28)
45°	0.385 (0.74, 0.57)	0.102 (0.49, 0.27)	0.124 (1.43, 0.60)	0.096 (0.49, 0.27)	0.789 (1.31, 0.71)	0.665 (1.19, 0.19)
60°	0.221 (0.91, 0.43)	0.047 (0.53, 0.18)	0.069 (1.46, 0.41)	0.044 (0.51, 0.15)	0.781 (1.34, 0.50)	0.444 (1.87, 0.50)

**Table 9:** The dimensionless maximum deflections of skew conoids (*hl*/*hh* = 0.25 and *a*/*h* = 10) subjected to sinusoidal hygrothermal distribution (Δ*T* = 300 °C, Δ*C* = 0.01%).

Skew Angle	0°/90°	45°/−45°	0°/90°/0°	45°/−45°/45°	0°/90°/90°/0°
0°	0.323	0.249	0.201	0.210	0.132
15°	0.266	0.264	0.172	0.188	0.125
30°	0.176	0.223	0.115	0.144	0.080
45°	0.084	0.132	0.104	0.080	0.108
60°	0.094	0.125	0.105	0.125	0.098

**Table 10:** The non-dimensional central deflections of laminated skew conoids subjected to sinusoidal hygrothermal.

Skew Angle	*a*/*h* = 5	*a*/*h* = 10	*a*/*h* = 20	*a*/*h* = 50	*a*/*h* = 100
0°	7.742	6.527	4.878	4.241	4.849
15°	3.253	2.691	2.101	1.885	2.036
30°	1.084	0.888	0.697	1.031	1.799
45°	0.200	0.169	0.222	0.431	0.770
60°	0.102	0.095	0.104	0.145	0.220

**Table 11:** Effect of curvature (*hl*/*hh*) on the dimensionless maximum stresses of two-layer (0°/90°) conoids subjected to sinusoidal hygrothermal distribution. (Δ*T* = 300 °C, Δ*C* = 0.01%).

Stresses	*hl*/*hh*					
	0.25	0.20	0.15	0.10	0.05	0.00
$\bar{\sigma_{x}}$	−463.40	−486.05	−485.00	−445.01	−346.33	−170.05
$\bar{\sigma_{y}}$	−2738.92	−2569.06	−2381.98	−2172.71	−1935.42	−1663.12
$\bar{\tau_{xy}}$	3838.81	3786.63	3561.32	3055.54	2115.01	545.88
